# Evaluation of safety and efficacy of different protocols of collagen cross linking for keratoconus

**Published:** 2020

**Authors:** Tanu Singh, Mukesh Taneja, Somasheila Murthy, Pravin Krishna Vaddavalli

**Affiliations:** * Department of Ophthalmology, Government Medical College and Hospital, Chandigarh, India; ** L.V. Prasad Eye Institute, Telangana, India

**Keywords:** keratoconus, cornea, cross linking, CXL, accelerated CXL, hypo-osmolar riboflavin

## Abstract

**Introduction:** Collagen cross-linking is a well-established modality that could stop the keratoconus from progressing. Off late, newer protocols have been suggested for progressive keratoconus, which include the use of hypoosmolar riboflavin for thinner corneas and the use of accelerated CXL protocol to reduce the effective treatment time.

**Objective:** To assess the safety and efficacy of different protocols of conventional CXL, hypoosmolar CXL and accelerated CXL in patients with keratoconus.

**Materials & methods:** It was a prospective, interventional study with minimum of 12 months follow-up. Patients were divided into 3 groups; conventional CXL, CXL using hypotonic riboflavin and accelerated CXL group. Primary outcome measures - For efficacy, Sim Kmax and Sim Kmin (Progression (Kmax > +1 D), stabilization (Kmax +1 D to -1 D) and regression (Kmax > -1 D). For safety - endothelial count evaluation (decrease >10% amounted to compromise the safety of the procedure). Secondary outcome measures - BCVA and adverse events.

**Results:** 32 eyes underwent isotonic CXL treatment. Pre-treatment and post-treatment BCVA were 0.16 +/- 0.15 and 0.10 +/- 0.11 log MAR; specular counts 2782.81 +/- 307.25 (cells/ mm2) and 2708.5 +/- 263.27 (cells/ mm2) (p=0.05); KMax values 55.31 +/- 4.12 D and 53.9 +/- 3.77 D (p=0.0001).

16 eyes underwent hypotonic CXL treatment. Pre-treatment and post-treatment BCVA were 0.15 +/- 0.13 log MAR and 0.14 +/- 0.14; specular count 2701.19 +/- 243.25 (cells/ mm2) and 2713.5 +/- 369 (cells/ mm2) (p= 1) and KMax values 54.74 +/- 7.44 D and 52.74 +/- 6.76 D (p = 0.002).

15 eyes underwent accelerated CXL treatment. Pre-treatment and post-treatment BCVA were 0.16 +/- 0.15 and 0.10 +/- 0.12 log MAR; specular counts 2967.53 +/- 356.48 and 2893.07 +/- 336.55 (cells/ mm2) (p = 0.78) and KMax values 55.19 +/- 5.46 D and 54.24 +/- 5.33 D (p = 0.337).

**Conclusion:** All three protocols appeared safe and efficacious as therapeutic regimen for progressive keratoconus.

## Background

Keratoconus is a clinical condition associated with conical shape of the cornea due to its thinning and subsequent protrusion [**1**]. The process is described as non-inflammatory and progressive, without cascade of cellular infiltration and vascularization.

Treatment options available for keratoconus include spectacle corrections, rigid contact lenses, and intracorneal ring segments as a means to improve the vision and rehabilitation [**2**-**4**]. The resistant disease that continues to progress, requires lamellar or penetrating keratoplasty as the potential treatment modalities [**5**,**6**]. In recent years, the goal of keratoconus treatment also included prevention of further disease progression, beside the improvement in visual acuity of the patients. The latest treatment modality that focuses on the corneal disease pathology and biomechanically increases its rigidity, thereby preventing the advance in disease severity, is cross-linking (CXL) of the corneal collagen.

This technique was first described in Dresden in Germany and it includes photo polymerization of collagen fibers of the stroma, which is induced by the combination of a photo-sensitizer (Vitamin B2/ Riboflavin) and ultraviolet rays (UV) [**7**,**8**]. These rays increase the quantity of intra-fibrillar and inter-fibrillar covalent bonds, thereby increasing the stiffness of the cornea and in this process, also enhancing the resistance of collagen against degradation by enzymes [**9**,**10**]. For safety purposes, the minimum corneal thickness of 400 microns after epithelial removal is necessary for the effective treatment of keratoconus by CXL [**11**]. This is quantified by pachymetry and this limit has been established in order to avoid endothelium damage.

However, in progressive stages of keratoconus, thinning of the cornea, causes a residual stromal thickness lesser than the minimum of 400 microns. So, the efficacy of CXL in such cases remains unclear. Hypoosmolar Riboflavin solution has been shown to increase the pretreatment thickness of such corneas, by Hafezi and associates, after which CXL could be used for further treatment [**12**].

The standard CXL therapy requires a minimum of 1 hour of exposure time for the patients. At present, only Dresden’s protocol has documented safety and efficacy profile for corneas with thickness >400 microns using this therapy. Nowadays, accelerated CXL has been described as another mean of fast-tracking the process with higher irradiance, thereby decreasing the overall period of exposure [**13**]. 

Safety and efficacy regarding the use of this accelerated CXL therapy in corneas thinner than 400 microns has not been documented yet. Whether or not this accelerated therapy can work for such corneas pretreated with hypoosmolar Riboflavin has not been clearly documented. The present study was conceptualized to evaluate the outcomes of accelerated CXL in such cases of keratoconus. 

## Materials and methods

The present study is a prospective, non-randomized, interventional case series conducted at a tertiary eye care hospital in India. The patients were enrolled over a period of 12 months and then followed up over another 12 months. Approval was taken from the institutional ethics committee and the study was performed in accordance to Helsinki Declaration.

The patients were included as per predefined criteria of inclusion and exclusion. All patients, regardless of their sex, who were 12 years old or older, having mild to moderate progressive keratoconus (defined as an increase in Sim K values by 0.5 D in 6 months or 1 D in 1 year) with BCVA 20/ 40 and a minimum follow-up of one year, were included in the study.

Exclusion criteria included patients who had ocular pathologies like vernal keratoconjunctivitis, corneal scar, corneal dystrophy, corneal degeneration and corneal hydrops. Patients having inflammatory conditions, posterior segment pathologies, ultrasonic pachymetry <350 microns and endothelial cell count <2000 cells/ sq mm were also excluded. In addition, all patients with systemic diseases like diabetes, thyroid dysfunction, collagen-vascular disorders and auto-immune disorders were not included. After informing the patients about the standard and the other modified protocols available for treating keratoconus, an informed consent was taken and patients were allocated into their respective groups. Patients having a corneal thickness of >400 microns were given a choice between the standard and the accelerated protocols, while those having a pachymetry of <400 microns were treated with hypotonic riboflavin only.

Finally, patients were divided into three groups:

• Eyes undergoing conventional CXL procedure using Indian made 0.1% isotonic riboflavin (K-Link Riboflavin, Appaswamy Ocular Devices, Puducherry) (epithelial debridement with 3mW UVA exposure for 30 minutes), in cornea with thickness of more than 400 microns after epithelial debridement.

• Eyes undergoing CXL procedure using hypotonic riboflavin i.e. riboflavin without dextran (Appaswamy Ocular Devices, Puducherry), in cornea with thickness of less than 400 microns after epithelial debridement.

• Eyes undergoing accelerated CXL procedure using 0.1% Riboflavin with 20% dextran (Avedro Inc., Massachusetts, USA) and KXL (epithelial debridement with 18mW UVA exposure for 5 minutes), in cornea with thickness of more than 400 microns after epithelial debridement.

The assessment was done at baseline and at 1, 3, 6 and 12 months after intervention; it included UCVA (log MAR units), BCVA (log MAR units), examination under the slit-lamp, ultrasound pachymetry (Pachy Meter SP3000; Tomey, Nagoya, Japan), video keratography (Orbscan II; Bausch and Lomb Surgical), measurement of the intra-ocular pressure with Goldmann tonometer (Haag-Streit AG, Koeniz, Switzerland) and evaluation of the endothelial cells with the Tomey EM-3000, Nagoya, Japan. Unmasked analysis was fine for all acquired images. 

Reliability of the video keratography measurements, was improved by performing at least 3 consecutive ones for all eyes. If the value of Kmax differed by more than 1 D among the 3 scans, 2 more scans were further done. The scan with the median Kmax value was finally analyzed after all the visits.

## Methodology

GROUP 1 (**Isotonic CXL**): After achieving surface anesthesia with 0.5% Paracain eye drops, mechanical debridement of corneal epithelium over a zone of 8 mm was done. 0.1% Riboflavin drops were instilled onto the cornea at a rate of 1 drop/ 2 minutes for 30 minutes, followed by UV irradiation exposure at 370nm/ sec for 30 minutes. Instillation of 0.1% Riboflavin drops was continued during irradiation.

GROUP 2 (**Hypotonic CXL**): All the steps were the same as for conventional CXL, however, instead of isotonic riboflavin, the cornea was initially saturated with hypoosmolar riboflavin by pouring hypoosmolar riboflavin over the cornea, drop by drop, at every two minutes, followed by irradiation for 30 minutes. 

GROUP 3 (**Accelerated CXL**): Calibrated KXL insert with independent power meter provided for the delivery of 18 mW/ cm2 was selected and the cornea was exposed for 5 minutes after an initial soakage of 30 mins.

During any of the procedures, if intra-op pachymetry values went below 350 microns, as a safety precaution, distilled water was instilled drop by drop for 2 minutes to swell up the cornea beyond 400 microns, and then the procedure was continued as per protocol.

Postoperatively, topical antibiotics were given 4 times daily for 7 days. Low potency steroid drops were started 4 times daily once the epithelium healed and tapered off over a month’s time. In addition, tear substitute drops were given 4 times daily for one month.

**Outcome Measures**

**Primary Outcome measures**, regarding the efficacy, were evaluated using Sim Kmax and Sim Kmin on topography with Orbscan to check for progression (Kmax > +1 D), stabilization (Kmax +1 D to -1 D) and regression (Kmax > -1.0 D). For safety, endothelial count was taken into consideration and any decrease in endothelial cell count >10% was considered significant and amounted to compromise in the safety of the procedure.

**Secondary outcome measures** were BCVA and occurrence of any adverse events (e.g. epithelial defects, corneal edema, corneal haze, keratitis, scarring, and cataract).

**Statistical Analysis**

Calculation of the sample size was done to detect a difference of 4% between the pre-op and post-op mean topographic value (Kmax). This was done at a significance level of 0.05 and 80% study power, assuming 4.1% of standard deviation. An anticipated loss to follow-up rate of 10% gave a final sample size of 15 in all the groups. Statistical analysis was done with the R software (version 2.12). The mean difference between the pre-op and follow-up time point scans of corneal topography were compared by multiple comparisons of means with Dunnett Contrasts from a mixed effect model, and adjusted p value was obtained by Bonferroni method. The mean difference between the procedure at pre-op and at 12 months for Specular and for Kmax was computed by using multiple comparisons of means with Tukey Contrasts from a linear model, the adjusted p value being obtained by Bonferroni method. Comparison between the pre-op and 12 months median BCVA was done using Wilcoxon signed-rank test.

## Results

A total of 74 eyes underwent CXL over a period of 12 months, of which 37 were in the isotonic CXL group, 19 in the hypotonic CXL group and 18 in the accelerated CXL group. However, 11 eyes were excluded from the study as they could not be followed-up and finally 63 eyes (32 in isotonic, 16 in hypotonic and 15 in accelerated CXL group) were taken into consideration for analysis. 

**Fig. 1:**
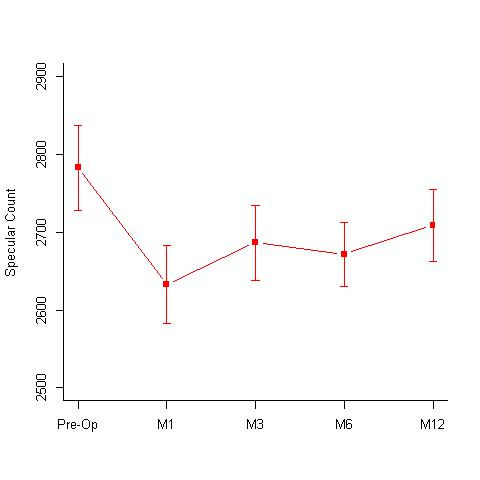
Specular counts (cells/ mm2) seen at 1st, 3rd, 6th and 12-months follow-up in the isotonic CXL group. Pre-operative specular count was 2782.81 +/- 307.25 (cells/ mm2) and 2708.5 +/- 263.27 (cells/ mm2) at 12 months (p=0.05) M = months

**Fig. 2:**
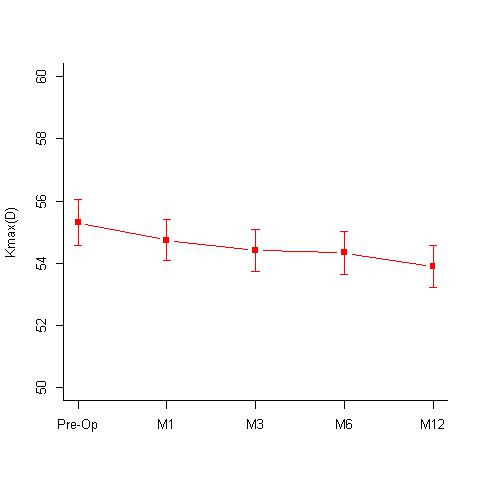
Mean KMax values [Diopters (D)] seen at 1st, 3rd, 6th and 12-months follow-up in the Isotonic CXL group. Mean KMax values pre-operative was 55.31 +/- 4.12 D and 53.9 +/- 3.77 D at 12 months (p=0.0001) M = months; D = diopters

**Isotonic group:** A total of 32 eyes underwent isotonic CXL treatment. The group had a mean age of 15.72 +/- 3.59 years. The mean pre-treatment and post treatment BCVA values at 12 months, were 0.16 +/- 0.15 and 0.10 +/- 0.11 log MAR; specular counts were 2782.81 +/- 307.25 (cells/ mm2) and 2708.5 +/- 263.27 (cells/ mm2) (p=0.05) (**Fig. 1**); KMax values were 55.31 +/- 4.12 D and 53.9 +/- 3.77 D (p=0.0001) (**Fig. 2**); KMin values were 48.59 +/- 4.15 D and 47.98 +/- 3.75 D and the pachymetry values were 438.44 +/- 31.16 microns and 426.09 +/- 47.07 microns, respectively. Out of 32 eyes, 16 eyes showed disease regression, 15 eyes were stable, while 2 eyes showed worsening.

**Fig. 3:**
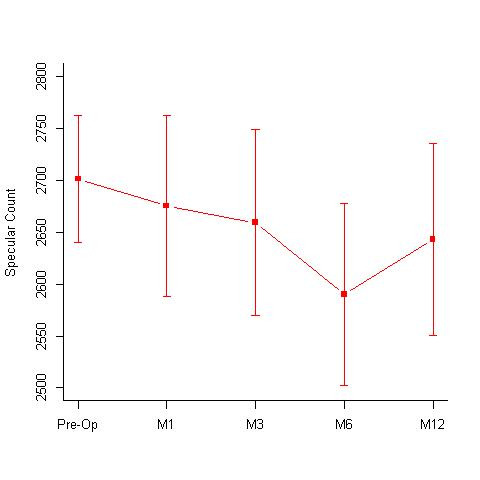
Specular counts (cells/ mm2) seen at 1st, 3rd, 6th and 12-months follow-up in the Hypotonic CXL group. Pre-operative specular count was 2701.19 +/- 243.25 (cells/ mm2) and 2713.5 +/- 369 (cells/ mm2) at 12 months (p= 1) M = months

**Fig. 4:**
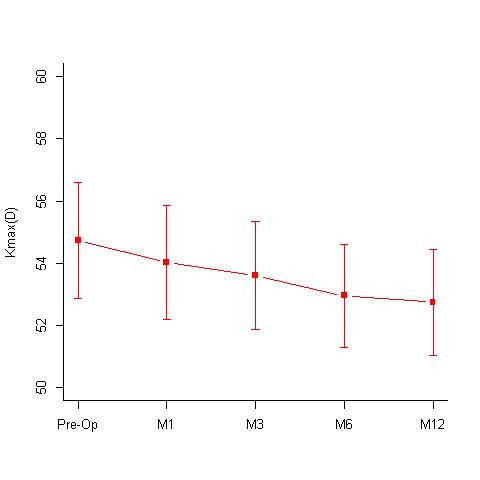
Mean KMax values [Diopters (D)] seen at 1st, 3rd, 6th and 12-months follow-up in the Hypotonic CXL group. Mean KMax values pre-operatively were 54.74 +/- 7.44 D and at 12 months 52.74 +/- 6.76 D (p = 0.002) M = months; D = diopters

**Hypotonic group:** A total of 16 eyes underwent hypotonic CXL treatment. The group had a mean age of 16.44 +/- 4.53 years. The mean BCVA pre-treatment and post treatment at 12 months were 0.15 +/- 0.13 log MAR and 0.14 +/- 0.14; the mean specular count was 2701.19 +/- 243.25 (cells/ mm2) and 2713.5 +/- 369 (cells/ mm2) (p= 1) (**Fig. 3**) and the mean KMax values were 54.74 +/- 7.44 D and 52.74 +/- 6.76 D (**Fig. 4**) respectively. The treated eyes showed statistically significant improvements with the Kmax flattening by 1.99 D at 12 months; p value of 0.002. The mean KMin values pre- and post-treatment were, 48.57 +/- 5.84 D and 47.61 +/- 6.48 D respectively and the mean pachymetry values were 397.06 +/- 9.82 microns and 371.06 +/- 14.64 microns respectively.

**Fig. 5:**
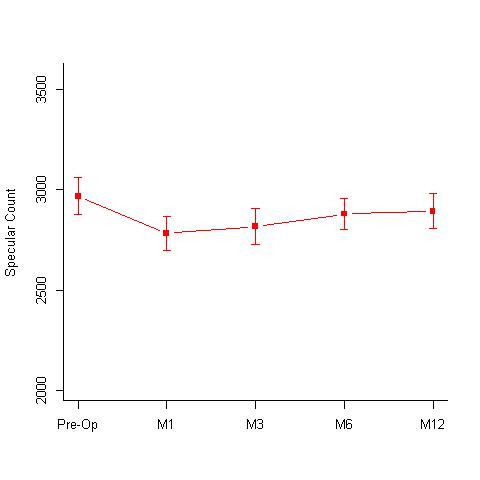
Specular counts (cells/ mm2) seen at 1st, 3rd, 6th and 12-months follow-up in the Accelerated CXL group. Pre-operative specular count was 2967.19 +/- 356.48 (cells/ mm2) and 2893.07 +/- 336.55 (cells/ mm2) at 12 months (p= 0.78) M = months

**Fig. 6:**
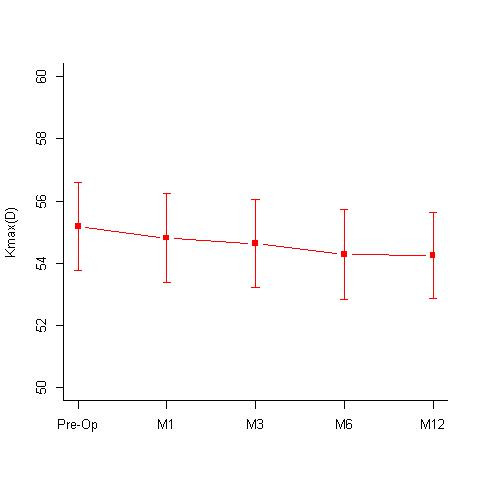
Mean KMax values [Diopters (D)] seen at 1st, 3rd, 6th and 12-months follow-up in the Accelerated CXL group. Mean KMax values pre-operatively were 55.19 +/- 5.46 D and at 12 months 54.24 +/- 5.33 D (p = 0.337) M = months; D = diopters

**Accelerated group:** A total of 15 eyes underwent accelerated CXL treatment. The group had a mean age of 14.07 +/- 2.71 years. The mean BCVA, pre- and post-treatment at 12 months were 0.16 +/- 0.15 and 0.10 +/- 0.12 log MAR; the mean specular counts were 2967.53 +/- 356.48 and 2893.07 +/- 336.55 (cells/ mm2) (p = 0.78) (**Fig. 5**) and the mean KMax values were 55.19 +/- 5.46 D and 54.24 +/- 5.33 D respectively (**Fig. 6**). There was a decrease in Kmax values by about 0.95 D. However, the difference was not statistically significant (p = 0.337). The mean KMin values, pre- and post-treatment were 48.89 +/- 4.65 D and 48.75 +/-4.49 D and the mean pachymetry values were 384 +/- 18.94 and 390.07 +/- 28.74 microns, respectively.

**Fig. 7:**
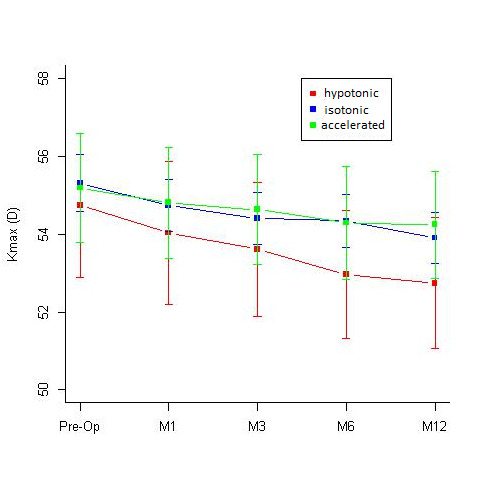
Comparative assessment of the mean KMax values [Diopters (D)] seen at 1st, 3rd, 6th and 12-months follow-up in the Hypotonic, Isotonic and Accelerated CXL group

**Fig. 8:**
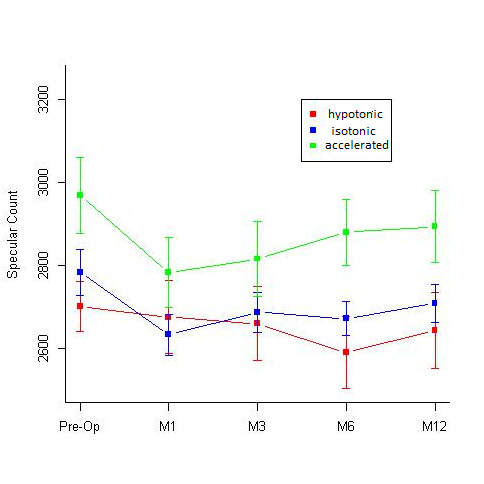
Comparative assessment of the specular counts (cells/ mm2) seen at 1st, 3rd, 6th and 12-months follow-up in the Hypotonic, Isotonic and Accelerated CXL group

A comparative assessment of the Kmax values at different time intervals done in the 3 groups revealed a statistically significant flattening of the cornea in isotonic and hypotonic CXL groups, while the decrease in Kmax value in the accelerated group was not statistically significant (**Table 1**, **Fig. 7**). While comparing the change in the specular count in the 3 groups, none of them showed any significant drop in the specular count at 12 months. The accelerated group showed a significant drop in the endothelial count at the 1st and the 3rd month of the follow-up, however the counts recovered by 12 months and were comparable to the pre-operative values (**Table 2**, **Fig. 8**).

**Table 1:** Difference in the Kmax values at 1st, 3rd, 6th and 12-months follow-up when compared to the pre-operative Kmax values in the hypotonic, isotonic and accelerated CXL groups

	Hypotonic		Isotonic		Accelerated	
	Estimate	P-value	Estimate	P-value	Estimate	P-value
1st month	-0.7062	0.88	-0.57	0.0496	-0.37	1
3rd month	-1.125	0.20	-0.90	0.00028	-0.56	1
6th month	-1.7812	0.0079	-0.97	0.0002	-0.9	0.402
12th month	-1.9937	0.002	-1.41	0.0001	-0.95	0.337

**Table 2:** Difference in the specular count at 1st, 3rd, 6th and 12-months follow-up when compared to the pre-operative specular count values in the hypotonic, isotonic and accelerated CXL groups

	Hypotonic		Isotonic		Accelerated	
	Estimate	P-value	Estimate	P-value	Estimate	P-value
1st month	-26	1	-150.16	0.07	-184.67	0.01
3rd month	-41.81	1	-96.12	0.21	-152.27	0.03
6th month	-111.12	0.27	-111.22	0.14	-88.27	0.5
12th month	-58	1	-74.25	0.11	-74.47	0.78

Best corrected visual acuity in all three groups showed a statistically significant improvement in all three groups at 12 months follow-up (**Table 3**).

**Table 3:** Median BCVA (inter-quartile range) in hypotonic, isotonic and accelerated CXL group at 12 months follow-up

	Pre-op	12 months	P value
Hypotonic	0.18 (0-0.3)	0.1 (0-0.18)	<0.05
Isotonic	0.1 (0-0.3)	0.1 (0-0.18)	0.005
Accelerated	0.1 (0-0.3)	0.0 (0-0.18)	<0.05

Adverse events: 5 patients in the isotonic group and 2 from the accelerated CXL group developed significant corneal haze after the procedure. While, 1 patient in the hypotonic group and 1 in the isotonic group developed sterile infiltrate.

## Discussion

CXL has come up as one of the most crucial therapeutic modalities for advanced keratoconus. There has been a rapid rise in the number of publications in the literature on CXL, ever since it was first reported by Spoerl et al. in 1998 [**8**]. However, there remains a deft of strong evidence in support of extensive usage of this modality, despite an ever-increasing description in literature. The present study reports the 12-months post therapy results of CXL, and demonstrates a statistically significant improvement in Kmax values, taking into account the specular count, that, at no point in time, showed a decrease of more than 10% from the baseline, thus, rendering the procedure to be safe. We included a total of 74 patients of whom 63 completed the 12 months follow up. Our first group had a total of 32 eyes, which underwent standard CXL using Isotonic riboflavin, and these patients showed a significant difference in Kmax values, without any significant decrease in the specular count. Similar results were shown by previous reported studies. 

In 2008, Snibson et al. reported that 9 eyes showed improvement (improved Kmax with a mean value of -0.74 D) after 1 year. In their 11 control eyes, the disease showed progression; mean Kmax of -1.28 D [**14**]. O’Brart et al. showed an average improvement in the value of Orbscan simulated keratometry by -0.62 D, after follow up of 1.5 years in the treatment group; P value <0.001. On the other hand, there was progression in the eyes of the control group by a mean of -0.14 D; P value < 0.3 [**15**]. In a randomized controlled trial with 1-year follow-up of treatment group and 3 months follow-up for control eyes, Hersh et al. reported an improvement in the Kmax (by mean of -2.0 D) from baseline in the treatment group. However, they reported no change in Kmax for control eyes at 3 months [**16**]. Several other studies have reported good outcomes with this technique. Caparossi et al. reported decreased mean K value in 44 eyes, by -2.24 D [**17**]. Raiskup-Wolf et al. documented 33 eyes with reduction in Kmax by a mean of -2.57 D [**18**]. 

The major drawback for CXL is the requirement of at least 400 microns, including a margin of safety, of the stromal thickness (excluding the epithelium of corneal). It is well described that in the advanced stages of keratoconus, there is increased thinning of the cornea, which more than often reduces the stromal thickness to less than 400 microns. Kymionis et al. studied the effect of Vitamin B2 and UV-A rays in thin cornea, using Dresden’s protocol in 12 eyes and concluded that there were no immediate intra and post-operative complications, but there was a significant endothelial damage [**19**]. This made researchers look for a safer option in the thin corneas [**12**]. Pre-operatively, the usage of hypo-osmolar Vitamin B2 has been described to influence swelling of the thin corneas with a stromal thickness of at least 400 microns. This has shown no complications post-operatively. 

In a similar study performed on 32 eyes, Raiskup et al. observed stability of keratoconus one year after the cross-linking procedure using hypoosmolar riboflavin [**20**]. Thus, the use of hypoosmolar riboflavin helped in the application of CXL even to those corneas that could potentially be ineligible because of they had less than the minimum required thickness. Using the same protocol as established for the treatment of thin corneas, Hafezi et al. performed CXL in a patient with a corneal thickness of 268 microns [**21**]. There was a distinct progression of 1.9 D at 3 months and 2.3 D at 6 months respectively. With this, the authors concluded that a minimal pre-op stromal thickness of 330 microns needs to be respected for successful CXL procedure in thin corneas. In their study, Kaya et al. compared the thickness of cornea using an iso-osmolar riboflavin solution plus 20% dextran with a hypoosmolar riboflavin solution with no dextran [**22**]. The authors concluded that hypoosmolar riboflavin leads to a significant swelling effect during the procedure, however, the iatrogenic swelling effect might be short acting and not durable throughout the UVA application; thereby, doubting the safety and warranting the need for continuous pachymetry measurements during the procedure. 

In the present study, a total of 16 patients underwent CXL using hypotonic riboflavin. A statistically significant improvement was noted in the treated eyes with a flattening of Kmax by 1.99 D at 1 year, with a p value of 0.002. No significant difference in the endothelial cell density was found at any point when compared with the baseline (p=1). These results were consistent with the results published in the past.

A third protocol with accelerated CXL has been described recently, as an alternative stimulator of the procedure by using higher irradiance to the patients in order to decrease the time of exposure to radiation. Alternative photoactive cross-linking agents, effective with much abbreviated UV-A exposures can be used to achieve the reduction in exposure time. In the present study, we also tested this protocol. Out of a total of 18 patients, 15 completed the 12-months follow-up. At the end of the 12-months follow-up period, the mean K-value showed a decrease of 0.95 D from the mean baseline pre-op values. In spite of this difference of not being statistically significant (p=0.337), the procedure certainly helped stabilizing the progression of the disease. The procedure showed a significant decrease in the endothelial count on the 1st and the 3rd month follow-up, however, at the end of the 12-months period, the endothelial count improved and the difference was not significant when compared to the baseline (p=0.78). The endothelial count was at no point less than 10% of its baseline values, thereby not compromising the safety of the procedure. 

In a randomized study of 138 eyes with keratoconus that underwent crosslinking at radiance of 3, 9, 18 or 30mW/ cm2, Shetty et al. observed that while there was an improvement in the corrected distance visual acuity in all groups at 1 year, the change was not significant in the 30mW/ cm2 group and most of the improvements occurred in the group with 18mW/ cm2 radiance. They also noted that the flattening effect of crosslinking was reduced with higher irradiation and shorter treatment duration [**23**]. Few other studies have reported a decrease in the spherical equivalent and cylinder error in both accelerated and conventional crosslinking, but with no significant difference between the 2 groups [**24**-**27**].

Therefore, our study is unique, evaluating all the described protocols for CXL treatment in keratoconus.

There were several transient complications we encountered in our patients. Postoperative haze was one of the major adverse effects seen after the procedure. 5 patients in the isotonic group and 2 from the accelerated CXL group developed significant corneal haze after the procedure. However, it was transient and responded well to low dose steroids. In their study, Caparossi et al. observed stromal haze in 9.8% of the eyes after CXL [**17**]. In contrast, in the present study, a mild degree of haze was observed in all the patients undergoing CXL, which resolved with time. 

Sterile infiltrates have been described in 7.6% of the treated eyes [**16**]. We encountered 2 cases of clinically significant sterile infiltrates postoperatively; one was associated with corneal oedema and an infiltrate paracentrally; the second eye developed subepithelial infiltrates (1 patient in the hypotonic group and 1 in the isotonic group). The lesions healed completely with scarring, which were away from the visual axis, and showed no effect on the visual acuity.

Keratoconus runs a variable course with different grades of severity and rates of progression to advanced disease. The associated difficulties in measuring outcome parameters that are reliable and reproducible, make randomized level 1 studies essential in order to evaluate the efficacy of CXL. Long-term studies also become crucial, to monitor the persistence of the CXL effect over a long duration. Overall, the results of our trial of CXL continue to support its efficacy in progressive keratoconus, with an improvement in Kmax values at 12 months. Additionally, no significant difference was noted at any point during the follow-ups, in the density of endothelial cells when compared with the baseline values; validating all three protocols to be safe. The associated risks with the protocols are minor compared to the associated morbidity of the advanced disease. Our findings suggested that CXL must be considered as a treatment modality in progressive keratoconus and the efficacy of new accelerated protocols helped to fasten the process with an acceptable safety profile. Also, the new protocols appeared to be as efficacious as the conventional protocols in arresting the progression of keratoconus. 

## Conclusion

CXL is a reliable and time proven treatment modality for keratoconus. The newer protocols with hypoosmolar riboflavin and accelerated CXL can reliably be used with adequate outcomes, being at par with the conventional technique. It is a safe technique without any significant complications and with good patient acceptability.

**Conflicts of Interest**

Nil. 

**Sources of Funding**

Nil. 
